# Highlighting of quorum sensing *lux* genes and their expression in the hydrothermal vent shrimp *Rimicaris exoculata* ectosymbiontic community. Possible use as biogeographic markers

**DOI:** 10.1371/journal.pone.0174338

**Published:** 2017-03-22

**Authors:** Simon Le Bloa, Lucile Durand, Valérie Cueff- Gauchard, Josiane Le Bars, Laure Taupin, Charlotte Marteau, Alexis Bazire, Marie-Anne Cambon-Bonavita

**Affiliations:** 1 Ifremer, Centre Bretagne, Laboratoire de Microbiologie des Environnements Extrêmes, REM/EEP/LM2E, UMR 6197 Ifremer-CNRS-UBO, ZI Pointe du Diable, CS, Plouzané, France; 2 Université de Brest, Laboratoire de Microbiologie des Environnements Extrêmes, UMR 6197 Ifremer-CNRS-UBO, Technopôle Iroise, 4 place Nicolas Copernic, Plouzané, France; 3 CNRS, Laboratoire de Microbiologie des Environnements Extrêmes, UMR 6197 Ifremer-CNRS-UBO, Technopôle Iroise, 4 place Nicolas Copernic, Plouzané, France; 4 Université de Bretagne-Sud, EA 3884, LBCM, Rue de Saint Maudé, Lorient, France; UPMC, FRANCE

## Abstract

*Rimicaris exoculata* is a caridean shrimp that dominates the fauna at several hydrothermal vent sites of the Mid-Atlantic Ridge. It has two distinct and stable microbial communities. One of these epibiontic bacterial communities is located in the shrimp gut and has a distribution and role that are poorly understood. The second colonizes its enlarged gill chamber and is involved in host nutrition. It is eliminated after each molt, and has colonization processes reminiscent of those of a biofilm. The presence and expression of genes usually involved in quorum sensing (QS) were then studied. At four sites, Rainbow, TAG, Snake Pit and Logatchev, two *lux* genes were identified in the *R*. *exoculata* epibiontic community at different shrimp molt stages and life stages. RT-PCR experiments highlighted *lux* gene expression activity at TAG, Snake Pit and Rainbow vent sites. Their potential QS activity and their possible roles in epibiont colonization processes are discussed. Moreover, phylogenetic analysis has shown the presence of three clades for *luxS* (*Epsilonproteobacteria*) and four clades for *luxR* (*Gammaproteobacteria*) genes, each clade being restricted to a single site. These genes are more divergent than the *16S rRNA* one. They could therefore be used as biogeographical genetic markers.

## Introduction

Deep-sea hydrothermal ecosystems of the Mid-Atlantic Ridge (MAR) are characterized by high pressure, no light and low nutrient availability. The geochemical conditions vary depending on the nature of the crust rock crossed by the hydrothermal fluids [1–2]. These ecosystems are sustained by microbial chemosynthesis instead of photosynthesis. Hydrothermal vents harbor a dense and endemic fauna which forms symbiotic associations with chemosynthetic microorganisms. This is the case of *Rimicaris exoculata* [3] (*Decapoda*: *Alvinocarididae*), an endemic shrimp of the MAR. This crustacean represents the predominant macrofauna of some sites of the MAR, such as Rainbow, TAG, Snake Pit and Logatchev. Based on COI and microsatellite analyses, a single haplotype can be identified along the MAR [4–5]. The genus is also found on the Central Indian Ridge and Mid-Cayman Spreading Centre [6–7]. It forms dense and moving aggregates, located closely along chimney walls in the gradient between hydrothermal fluids and cold oxygenated ambient seawater, in a temperature range between 3°C and 25°C [8–10]. This shrimp is neither predatory nor necrophagous and lives in symbiotic association with two distinct microbial communities in what is known as ectosymbiosis as symbionts are always retrieved outside the host cells. One symbiotic community is located in the gut [11–12], between the microvilli of the digestive cells and shows no visible septum [13–14]. Four main lineages are identified whatever the specimens studied, related to *Epsilonproteobacteria*, *Gammaproteobacteria*, Mollicutes and Deferribacteres. Yet their relative abundance per location and role are still enigmatic. The second symbiotic population is located in the gill chamber [15–28]. *R*. *exoculata* has an enlarged and almost closed gill chamber with hypertrophied mouthparts, which favors the colonization of the internal surfaces of the lateral carapaces (branchiostegites, Br) and of the mouthparts (scaphognathites, Sc) by bacteria [17]. According to microscopic observation, the gill chamber filamentous microbial community is dense, complex, and highly organized, [18], [20], [21]. Several bacterial groups mainly affiliated to *Epsilonproteobacteria* and *Gammaproteobacteria*, but also to *Zetaproteobacteria* and other groups such as Firmicutes or CFB have been identified. Briefly, considering all studied sites, about five OTUs sharing 93.5 to 97.5% similarity, are related to the *Epsilonproteobacteria Sulfurovum* genus, five to other *Epsilonproteobacteria* lineages, and two last ones to *Gammaproteobacteria* lineages, one sharing 98 to 99% similarity is related to *Leucothrix mucor* [21], [24] and one to methanotrophic symbionts (MOX lineage, [26]). Using cloning and FISH analyses, Petersen and colleagues showed that one *Epsilonproteobacteria* OTU (or two for Rainbow) is clearly dominant per site, but a single OTU for *Gammaproteobacteria* (related to *L*. *mucor*) is retrieved as dominant whatever the site analyzed [24]. Despite a large microbial diversity in the surrounding environment [29] and a bacterial community switch occurring between the first stages and later stages of the *R*. *exoculata* life cycle [26], these epibionts are systematically retrieved in the gill chamber of all studied specimens all along the MAR [20–24]. In this epibiontic community, several autotrophic metabolisms co-exist [21], [28]. Briefly, using a metagenomic approach, Jan and colleagues showed that dominant *Epsilon* and *Gammaproteobacteria* lineages are able to use sulfur and hydrogen for autotrophic carbon fixation through rTCA and CBB cycles respectively. Some *Gammaproteobacteria* (called the MOX lineage) would be methanotrophic. In addition, the newly described *Zetaproteobacteria* would be able to oxidize iron for carbon fixation through CBB cycle [28]. Finally, a recent study demonstrated the trophic involvement of autotrophic gill chamber epibionts, showing transtegumental absorption of labeled microbial organic matter by the host [27]. Jan and colleagues raised the question of direct competition between co-occurring epibionts with similar processes, such as sulfur or hydrogen oxidation and carbon fixation. This would be prevented, thanks to subtle differences in the gill chamber that would provide sufficient niche differentiation for epibiont activities, allowing their stable co-occurrence [28] in time and space. Moreover, their involvement in detoxification processes was suggested because epibiont autotrophic metabolisms would have beneficial side effects chelating heavy metals, and converting hydrogen sulfides to sulfur or nitrite to dinitrogen [27–28]. All these results reinforced the idea of a complex stable symbiosis in *R*. *exoculata* with *Gammaproteobacteria* and *Epsilonproteobacteria* as the recurrent main lineages.

**Fig 1:**
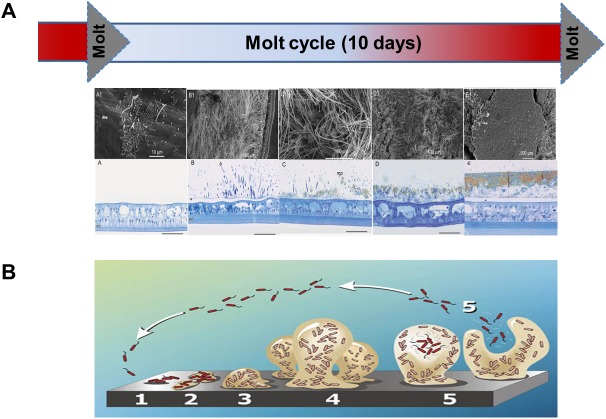
Acquisition of symbionts in the gill chamber of *Rimicaris exoculata*. (A) Epibiontic colonization through the molt cycle [22–23] compared to (B) biofilm formation [32].

Like all arthropods, *R*. *exoculata* molts. The microbial community of the gill chamber is eliminated at each molt, every 10 days [22], [23], [26] but not the digestive community as the gut is not subjected to the molt [13]. After each molt, epibionts re-colonize the host rapidly to form a new community, which develops through a series of different stages (Fig 1). First, this community attaches and grows as colonization spots showing organized single bacilli. Then these spots extend to rapidly form a dense microbial mat with long filamentous bacteria. This colonization is accompanied by accumulation of iron/sulfur oxides [22–23] until the next molt. Structure and establishment of this epibiontic community resemble those of a biofilm [30–32]. Based on substrate colonization experiments, Szafranski and colleagues suggested that these *R*. *exoculata* symbionts may be simple opportunists colonizing a new surface [33]. Putting aside the shrimp immune system that would probably prevent uncontrolled fouling [34], in biofilm formation whatever the surface colonized (living or not) bacteria can use a communication system dependent on their population density known as: the quorum sensing [35–41], (Fig 1). This QS enables biofilm formation including surface attachment, cellular arrangement and structural conformation. In a symbiotic community, this mechanism could help to maintain the biofilm attached to the host [42]. Using a 454 approach annotated by five individual taxonomic prediction tools [43], partial metagenomes were built and compared to databanks for taxonomic affiliation. Among these, *Epsilon* and *Gammaproteobacteria* taxobins were the most represented. One *luxS* gene and one almost complete *luxR* gene were retrieved in the *Epsilon* and *Gammaproteobacteria* taxobins respectively [28]. These could play a role in the shrimp colonization processes. QS systems can be divided into three primary classes based on autoinducer signal type and the means used for detection [38]. Typically, Gram-positive bacteria use peptide derivatives for communication, whereas Gram-negative bacteria use small diffusible molecules, *e*.*g*. *N*-acylhomoserine lactones (AHLs). However, Biswa and colleagues recently discovered the production of AHLs by a Gram-positive bacterium belonging to the *Exiguobacterium* genus, isolated from marine water [44]. The typical QS system of Gram-negative bacteria consists of a LuxI-like autoinducer synthase that produces constantly AHLs as signals. A LuxR-type receptor detects the AHLs when they exceed a threshold concentration controlling expression of specific genes [45]. Unfortunately, no *luxI* gene has ever been identified in our partial metagenomes [28]. A last class of QS system is a hybrid between the canonical Gram-negative and Gram-positive systems. This hybrid system was initially identified in the bioluminescent marine bacterium *Vibrio harveyi*, which produces and detects two distinct autoinducers, AI-1 and AI-2 [36]. In a similar way to other Gram-negative systems, AI-1 is an AHL [46], whereas AI-2 of *V*. *harveyi* is a furanosyl borate diester synthesized by the LuxS enzyme [47–48], with no resemblance to other autoinducers [49]. AI-2 is an interspecies communication molecule among bacteria [37] but has yet to be observed as an interkingdom one.

The process of colonization is strictly similar among all individuals and molts stages analyzed [22–23] reminiscent of a biofilm formation (Fig 1). Only two complete genes of QS (*luxS* and *luxR*) were revealed in the metagenome of the epibionts from Rainbow [28]. All this leads to the hypothesis that there could be a communication system and a strict control that allow an almost identical recolonization after each molt in terms of phylogeny and spatial distribution in the gill chamber. Moreover, even if several results demonstrate that bacterial AHL QS signals can also be sensed by eukaryotic organisms [50–52], the role of the QS in extreme environments has yet to be investigated in detail, particularly for hydrothermal vents [53]. Currently, nothing is known regarding the recognition pathways between *Rimicaris exoculata* and its two symbiotic microbial communities (gill chamber and digestive system) and between epibionts themselves. Therefore, this study was dedicated to analyzing i) whether *lux* genes are retrieved and expressed in each *Rimicaris exoculata* epibiont community collected from four MAR sites and ii) whether the *lux* genes could be used as new biogeographic tools. To address these questions, the present study uses molecular approaches to explore *lux* genes from the gill chamber epibiont communities at distinct molt stages, guts and eggs from four vent sites from north to south: Rainbow, TAG, Snake Pit and Logatchev.

## Materials and methods

### Shrimp collection, DNA and RNA extraction

*Rimicaris exoculata* were collected from four vent fields along the MAR using the Ifremer research vessel R/V *Pourquoi pas*? Different oceanographic cruises visited the different hydrothermal sites: SERPENTINE2007 sampled at Logatchev (14°45' N;-3010 m), BioBaz2013 at Rainbow (36°14' N;-2320 m), and BICOSE2014 http://dx.doi.org/10.17600/14000100 at TAG (26°08' N;-3640m) and Snake Pit (23°23' N;-3480m). The slurp gun of the remotely operated vehicle (ROV) Victor 6000 was used to collect the specimens. No specific permissions were required to collect these samples in international deep seawaters. The study did not involve endangered or protected species.

Once aboard, eight living juveniles and ten living *R*. *exoculata* specimens at different molt stages were immediately frozen at −80°C (to be dissected later at the laboratory) or dissected on board when possible to separate the mouthparts (scaphognathite) from the inner face of the gill chamber (branchiostegite), and to sample the stomach, the gut and the eggs if present. Then each part was frozen at -80°C. At the laboratory, these parts were used to extract DNA using the NucleoSpin Soil (Macherey-Nagel) kit according to manufacturer’s recommendations. The quality and size of the extracted DNA was assessed by 0.8% agarose gel electrophoresis. RNA was extracted with the NucleoSpin RNAII (Macherey-Nagel) kit and then treated using the Turbo DNASE kit (Ambion) to eliminate any traces of DNA. The concentration of DNA and of extracted RNA was estimated using an ND-1000 Spectrophotometer (Nanodrop) or the Qubit RNA HS kit using the Qubit 3.0 Fluorometer.

### Amplification of lux genes

Sequences corresponding to genes *luxS* and *luxR* were revealed in the metagenome of the ectosymbionts of the gill chamber [28], yet no primer was available in the literature. The two metagenome complete *lux* sequences were therefore used with BLAST to retrieve related *luxS* or *luxR* sequences available in the international data banks. Then, one alignment per gene was done using MAFT [54] with Geneious version 6.1.5 software. This made it possible to design new primers to try to amplify the two *lux* genes identified in the gill chamber epibiont metagenome (Table 1). The *luxS* and *luxR* genes were amplified from branchiostegites, scaphognathites, gut and stomach of two individuals at different molt stages from each sampled site and from eggs and juveniles (Table 2 and Table 3).

**Table 1:** Primers used (all sequences were designed during the study).

Gene	Name	Taxon targeted	Primer sequence	Amplicons size	Melting temperature
*luxS*	LuxS RexF	*Epsilonproteobacteria*	5’ATGCCATTATTAGATA3’	550 pb	44°C
	LuxS RexR		5’TTTTTTATTNGNGAGT3’		40°C
*luxR*	LuxR RexF	*Gammaproteobacteria*	5’ATGATAAACCTCGTTGCT3’	560 pb	51°C
	LuxR RexR		5’AGTTTTTACACAGCAATTAGAA3’		52°C

**Table 2:** *Rimicaris exoculata* samples used for amplification of *lux* genes indicating parts sampled from different vent sites.

		Vent sites			
Samples		Rainbow	TAG	Snake Pit	Logatchev
R. *exoculata* adults	branchiostegite	✓	✓	✓	✓
	scaphognathite	✓	✓	✓	✓
	gut	✓	✓	✓	✓
	stomach	✓	✓	✓	✓
juvenile		⦸	✓	✓	⦸
eggs		⦸	✓	✓	✓

Samples available (✓) or not (⦸) for this study.

**Table 3:** *Rimicaris exoculata* samples used for amplification of *lux* genes according to the molt stages.

	Vent sites			
Samples	Rainbow	TAG	Snake Pit	Logatchev
R. *exoculata* adults	beginning, middle and end of molt cycle	beginning and end of molt cycle		

For each site and each stage of molt or age, two specimens were analyzed. Reaction mixtures for PCR amplification contained 100 ng template DNA, 20 pmol of each primer, 0.4 μmol of each deoxynucleotide triphosphate, 1X Go Taq Flexi green buffer (Promega), and 5U GoTaq Flexi polymerase (Promega). The final volume was adjusted to 25 μl with sterile water. The PCR program involved an initial denaturation step at 95°C for 5 min, followed by 30 cycles of 95°C for 1 min, 42°C or 50°C for 1.5 min according to the primer (Table 1), and 72°C for 2 min, with a final elongation step at 72°C for 10 min. After gel verification, PCR products from each replicate were pooled and then purified with the NucleoSpin Gel and PCR Clean-up kit (Macherey-Nagel). The size of fragments was determined using Smart Ladder markers of 10,000 bp and 1000 bp (Eurogentec). The amplification by RT-PCR was done with the OneStep kit (Qiagen). DNA presence was first tested for by PCR amplification on RNA extracts and gave no amplification. RNA extracts were normalized at 2μg/μL. Then 1 μL of RNA was used in 50μL mix to be retro-transcribed (30 min at 50°C) and amplified according to the following conditions: polymerase activation 15 min at 95°C; 40 cycles of 95°C for 1 min, 42°C or 50°C for 1.5 min, 72°C for 1.5 min, and a final elongation step at 72°C for 8 min. Scaphognathite RNA, extracted from a shrimp at the end of a molt cycle, was used as a positive control for amplification using 16S rDNA gene primers (E8F/U1492R (respectively 50-AGAGTTTGATCATGGCTCAG-30 and 50-GTTACCTTGTTACGACTT-30, 1484 bp, annealing temperature 49°C,- [55].

### Cloning of *lux* genes and phylogenetic analysis

For gill chamber samples, purified PCR products were used for direct sequencing to confirm primer efficiency and specificity. All samples were also cloned using the TOPO-TA kit (Invitrogen, Carlsbad, CA, USA). The insert size of positive *Escherichia coli* colonies was tested for by PCR screening with vector primers M13F and M13R. Then, several clones (Table 4) were sequenced by GATC Biotech (https://www.gatc-biotech.com/) according to the Sanger method [56] on a 3730xl ABI (Applied Biosystems) with Dye Deoxy ™ Terminator technology. Related sequences were then retrieved by comparing our sequences with those present in international databases using the BLAST tool [57], via the KoriBlast software (Korilog). Sequences were imported into Geneious version 6.1.5 software (Biomatters, available from (http://www.geneious.com/) and aligned with MAFT [54].

**Table 4:** Clone library for the *lux* genes of *Rimicaris exoculata* epibionts.

	Rainbow		TAG		Snake Pit		Logatchev	
Samples	*luxS*	*luxR*	*luxS*	*luxR*	*luxS*	*luxR*	*luxS*	*luxR*
**Gill chamber (all):**	**105**	**154**	**86**	**91**	**107**	**88**	0	**77**
*beginning of molt*	*21*	*50*	*34*	*35*	*25*	*26*	0	*20*
*middle of molt*	*10*	*10*	*0*	*0*	*0*	*0*	0	*0*
*end of molt*	*74*	*104*	*52*	*56*	*82*	*62*	0	*57*
Digestive tract (gut and stomach)	20	20	20	20	20	20	0	20
eggs	0	10	0	10	0	10	0	48
juveniles	0	10	0	10	0	10	0	0
Male (gill chamber and digestive tract)	0	0	20	30	0	0	0	0
Total	125	194	126	161	127	128	0	145
Total per site	**319**		**287**		**255**		**145**	

### Bioinformatic studies

Protein alignments were performed using the Geneious version 6.1.5 program. The bioinformatic identification of LuxR solos were first based on the presence of the C-terminal “HTHLUXR” motif (SMART00421) using SMART7 software (Simple Modular Architecture Research Tool) [58] and BLAST software [59]. Protein domains were identified using SMART7 software and were identified with a maximal *p*-value of 2.54e^-5^ for the “HTHLUXR” motif (SMART00421) and of 8.61e^-10^ for the “REC” domain (SM00448). In the next step, the amino acid residues at the WYDPWG-motif positions in the signal-binding domain (SBD) of AHL-sensors were added as metadata layers.

**Sequences** are available at the EMBL under the number LT220912 to LT220957.

## Results and discussion

### *Lux* gene detection in the epibiont community

**Fig 2:**
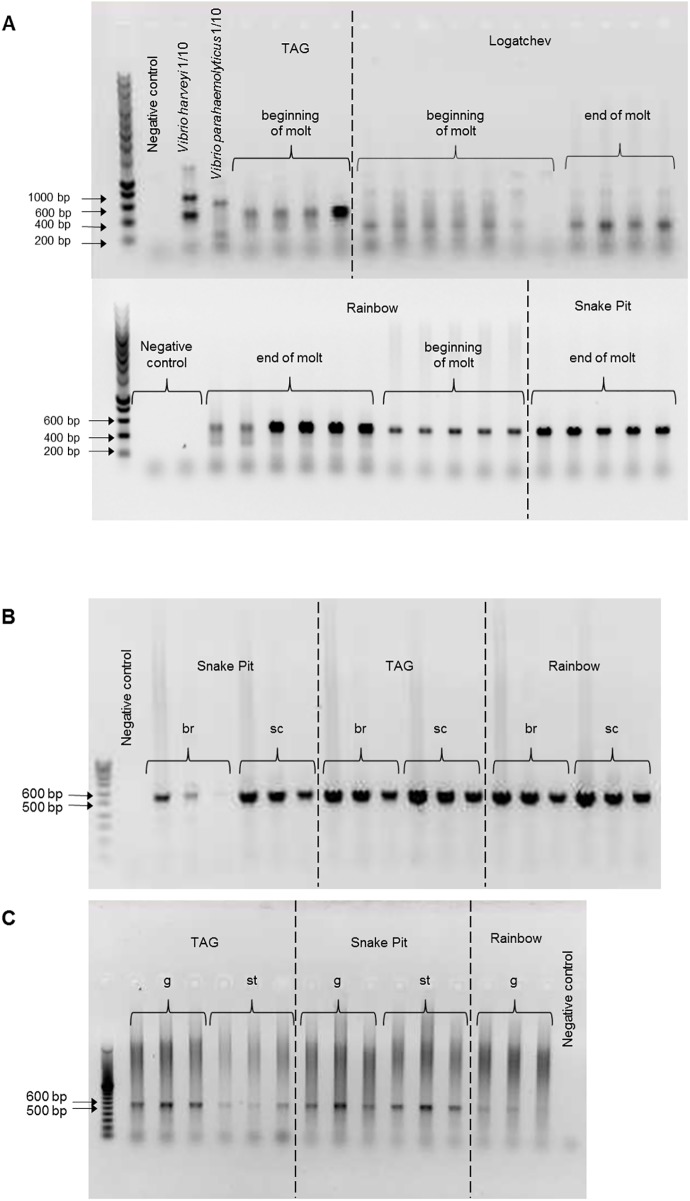
Example of PCR on gill chamber epibionts of *R*. *exoculata*. (A) *luxS* amplification. *luxS* genes from *Vibrio harveyi* and *Vibrio parahaemoliticus* were used as negative controls. (B) *luxR* amplification on branchiostegite (br) and scaphognathite (sc) epibionts. (C) *luxR* amplification on gut (g) and stomach (st) epibionts.

**Fig 3:**
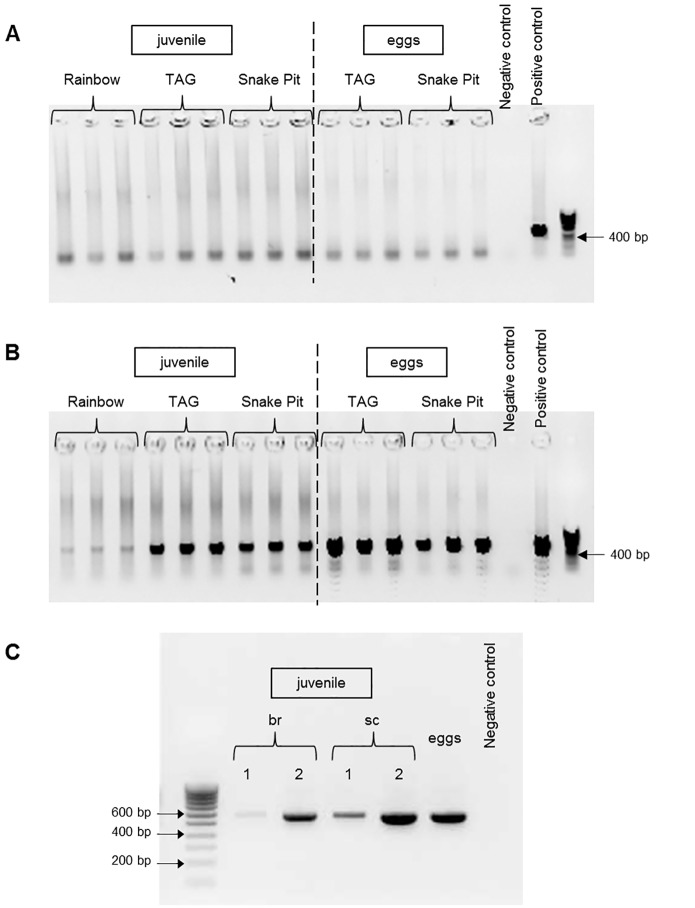
*lux* gene PCRs on epibionts of *R*. *exoculata* juveniles and eggs. (A) *luxS* amplification on scaphognathite and eggs. (B) *luxR* amplification on scaphognathite and eggs. (C) *luxR* amplification on (br) branchiostegite and (sc) scaphognathite at the beginning (1) and end (2) of the molt.

To detect the presence of *luxS* and *luxR* genes in the epibiont community according to metagenome data, PCR amplifications were done on DNA extracted from branchiostegites (Br), mouthparts (scaphognathites and exopodites (Sc)) and guts of shrimps from different vent sites (Rainbow, TAG, Snake Pit and Logatchev) at two or three molt stages (beginning, intermediate and end stages of the molt cycle) and on juveniles and eggs (Table 2 and Table 3, Figs 2 and 3). Both *luxS* and *luxR* have been correctly amplified, *luxR* always showed a better amplification.

We tested our *LuxS* primers on *Vibrio harveyi* and *Vibrio parahaemolyticus* to test their specificity. Several PCR products were obtained, but did not give the expected size. They were sequenced but were not affiliated to *luxS* genes (data not shown).

For the gill chamber epibionts (Br and Sc), the *luxS* genes were only amplified for adults from the Rainbow, TAG and Snake Pit sites (Table 5) showing better amplification levels at the end of the molt cycle (Fig 2). Direct sequencing on PCR products gave a single sequence related to *Sulfurovum sp*., (BLAST similarity levels of 75% for *luxS* nucleotide sequence), the closest related epsilonproteobacterial symbiont genus (92% for 16S rDNA gene *Sulfovorum* NBC37-1) [24]. Only non-specific amplicons were obtained for the sample from Logatchev, the oldest samples in our study (2007). This could be due to signal extinction caused by slightly lower DNA quality. This could also be due to unspecific amplifications. The sequencing of these cloned fragments confirmed the absence of any *luxS* gene amplification (data not shown). Although this could suggest that no *luxS* gene is present in the epibiont community of the gill chamber at this site, this seems unlikely as the epsilonproteobacterial symbiont diversity along the MAR always mainly cluster within *Sulfurovum sp*. group [24].

**Table 5:** PCR and RT-PCR (end of molt) amplification results per sample of different *Rimicaris exoculata* parts from different vent sites for the *luxS* gene analysis.

		Vent sites			
Samples		Rainbow	TAG	Snake Pit	Logatchev
R. *exoculata* adults	branchiostegite	✓	✓	✓	✕
	scaphognathite	✓	✓	✓	✕
	gut	✕	✕	✕	✕
	stomach	✕	✕	✕	✕
juvenile		⦸	✕	✕	⦸
eggs		⦸	✕	✕	✕

Inversely, the *luxR* gene was well amplified for all adult gill chamber samples whatever the vent site, including Logatchev (Table 6). PCR products were either directly sequenced or cloned and always gave a single sequence per site and per sample. The closest relative was a *Gammaproteobacteria*, *Osedax* symbiont and *Oleisprira* strain RB8 sp. sharing 80% and 77% similarity respectively for *luxR* nucleotide sequence, and 85% and 83% respectively for 16S rDNA gene using BLAST.

**Table 6:** PCR and RT-PCR (end of molt) amplification results per sample of different *Rimicaris exoculata* parts from different vent sites for the *luxR* gene analysis.

		Vent sites			
Samples		Rainbow	TAG	Snake Pit	Logatchev
R. *exoculata* adults	branchiostegite	✓	✓	✓	✓
	scaphognathite	✓	✓	✓	✓
	gut	✓	✓	✓	✓
	stomach	✓	✓	✓	✓
juvenile		⦸	✓	✓	⦸
eggs		⦸	✓	✓	✓

Positive PCR or RT-PCR are indicated by a (✓) and a negative PCR or RT-PCR are indicated by a (✕). Samples denoted (⦸) were not available.

DNA from eggs and juveniles gave no amplification for the *Epsilonproteobacteria* epibiont *luxS* gene, whereas it was amplified for the gill chamber epibionts of related adults. Inversely, the *Gammaproteobacteria luxR* gene gave good amplifications for all these samples. Surprisingly, for the egg samples, the main amplified product was sequenced but did not match any *luxR* gene and was unaffiliated in any database (data not shown). The faint band at the expected size was sequenced and revealed a *luxR* gene sequence. Regarding the first life stages, our results are in good agreement with previous studies using cloned sequences and FISH analyses of *R*. *exoculata* egg and juvenile epibionts [26]. Even though the same epibiont lineages are found throughout the shrimp life cycle, a switch between the main bacterial communities is observed. The first stages are dominated by the *Gammaproteobacteria*, and the later ones by the *Epsilonproteobacteria*. This may explain why *luxR* (*Gammaproteobacteria*) is sufficiently amplified, but not the *luxS* (*Epsilonproteobacteria*) during the first stages life of *R*. *exoculata*: the latter would be present in too low quantity.

Finally, DNA from the gut gave no amplification for the *luxS* gene. The epibiont community of the gut is mainly composed of lineages affiliated to Deferribacteres, Mollicutes, *Gammaproteobacteria* and *Epsilonproteobacteria*, but their relative abundance is still unknown [13–14]. These *Epsilonproteobacteria* are closely related to the gill chamber epibionts (99–100% of similarity for the *16S* rRNA gene) and so *luxS* would have been expected to be amplified using the same primers. DNA extracted from the gut is usually in low quantities and moreover, contains many host DNAs [13]. Here, DNA extractions lead to concentrations around 2.5 to 3.5 ng/μL. Moreover, inhibitors can be present (mainly minerals and organic matter), rendering it more difficult to amplify, even using *16S* primers [13]. Inhibition controls were performed by adding gut extracted DNA to a positive control DNA template. As PCR were still positive, the inhibition test was then negative. As shown by our amplification results (Fig 2A and 2B), *luxS* genes always gave faint amplifications. This inherent difficulty of amplification, together with the low level and quality of extracted DNA could have impaired the amplification of the specific *luxS* genes. In the gut, the *luxR* amplifications gave good results for all samples tested and all sequences were affiliated to the same *Gammaproteobacteria* as the gill chamber ones. As our *luxR* primers are specific to *Gammaproteobacteria*, this result could also suggest that they are more represented in the gut than *Epsilonproteobacteria*, for which our *luxS* primers are specific, as is the case for the first stages of life.

### QS and epibiont colonization

**Fig 4:**
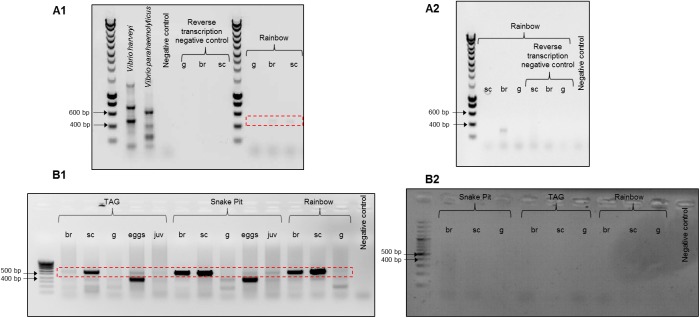
Example of *lux* RT-PCR on gill chamber epibionts of *R*. *exoculata*. Free RNAse/DNAse water was used as template for the negative control. (A) *luxS* amplifications were done on branchiostegite (br), scaphognathite (sc), and gut (g) shrimp epibionts at the end of molt cycle from Rainbow. A1 end of molt and A2 beginning of molt cycle (B) *luxR* amplification were done on branchiostegite (br), scaphognathite (sc), gut (g), eggs and juvenile (juv) epibionts at the beginning (B2) and at the end (B1) of the molt cycle. The dotted box indicates the correct PCR products size. B2 was more contrasted to try to observe any amplification.

The *luxS* and *luxR* transcripts were amplified by RT-PCR on all parts that were positively amplified in PCR (Table 5 and Table 6 and Fig 4). Despite the various dilutions of Br and Sc extracted RNA tried, no amplification was ever obtained for the Logatchev site samples, maybe because they were too old (SERPENTINE2007). For Rainbow, TAG and Snake Pit sites, RT-PCR amplicons were mostly obtained for shrimps at the end of the molt cycle. The sequencing of these fragments confirmed that it was the same *luxS* and *luxR* genes as those revealed by PCR for *Epsilon* and *Gammaproteobacteria*, respectively. However, it is surprising that the *luxS*/*luxR* expression was only detected only for the late molting stages. Indeed, QS systems appear to be involved in all phases of biofilm formation [60–61]. Our results could be explained by i) a denser epibiont community in the second part of the molt cycle that provided a greater quantity of extracted RNA, and/or ii) that QS expression occurs mostly when symbionts have almost completely colonized the gill chamber and start to regulate themselves to avoid invasion or competition. In model biofilm-forming bacteria, QS also contributes to the dispersal of biofilms [62]. QS could therefore be used here by symbionts just before the molt event to liberate some epibionts from the biofilm to prepare the new re-colonization afterwards.

*In silico*, the LuxS and LuxR protein sequence alignments show functional proteins with conserved domains essential for enzyme activities (see S1 File, S1 Fig and S2 Fig). The *luxS* gene transcripts were correctly amplified (RT-PCR) and the LuxS protein sequence is conserved and seems to be able to produce AI-2, still not identified in our study while shown to be stable over wide chemical ranges [63–64]. LuxS could therefore be involved in communication for our model.

No *luxI* has yet been found in the epibiont metagenome [28], and no AHLs could be detected in our study (see S1 File and S3 Fig). It is then possible that LuxR proteins retrieved in our study would be LuxR solos (S2 Fig) [65–67]. LuxR solos form a protein family highly similar to QS LuxRs, which does not possess an associated cognate LuxI protein. As the signal molecule capable of being perceived by the majority of LuxR solos is still unknown, they are potential candidates for the capture of a large number of bacterial or even eukaryote signaling molecules. In this way, they could be involved in interkingdom communications [68]. So the *lux*R gene expression detected here could synthesize LuxR homologs that could respond to exogenously produced AHLs made by other bacteria of the epibiont community or by compounds produced by the shrimp during colonization [50], [52], [69], [70]. These LuxR solos could therefore be part of another type of QS system. The, *lux* gene expression by the two main epibionts of the gill chamber observed on several vent sites and their conserved protein structure therefore suggest a potential *in situ* activity of QS implied in biofilm formation.

### Phylogenetic analysis: *luxS* and *luxR* as possible biogeographical markers

Functional genes, such as *pmoA* and *APS* can be used in phylogeny [21] and are usually more divergent than the 16S rDNA gene. Here our *lux* genes were proven to be transcribed and thus not to be pseudo genes subject to random mutation events. To determine whether the *luxS* and *luxR* genes could be used as biogeographical markers, 77 to 154 clones were sequenced from colonized gill chamber parts of *R*. *exoculata* at each vent site (Table 4). The sequences similarity level was 99.5% for the *luxS* gene and 99.8% for the *luxR* gene within each of the hydrothermal vent sites (Rainbow, TAG, Snake Pit and Logatchev). Conversely, sequence similarity level varied from 87.3% to 91.3% for the *luxS* gene and from 95.4% to 96.6% for the *luxR* gene between the different hydrothermal vent sites. Therefore, the *lux* genes sequence inter-site diversity was much greater than the intra-site diversity, clearly separating each site of origin. These *lux* genes were then used on all other samples (state of molt, juveniles, eggs, males, females, gut) to study the biogeography of epibionts from *R*. *exoculata*. All sequences clustered as a single clade per site, which was not the case with the 16S rRNA genes where Rainbow was split into two clades and TAG and Snake Pit were not clearly differentiated [24], [26]. So, these *lux* genes appear to be good biogeographical markers of the different studied sites.

**Fig 5:**
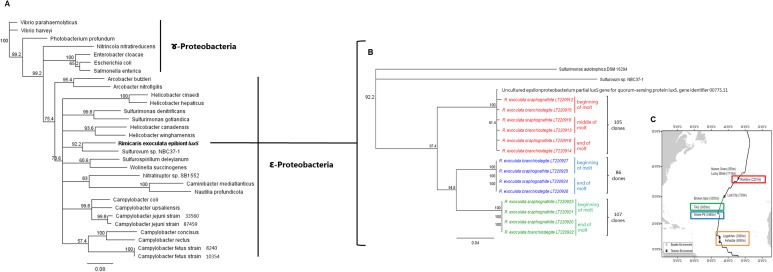
*luxS* gene phylogeny (calculated on 550 bp) of symbionts associated with the gill chamber of *R*. *exoculata*. The robustness was tested using 500 bootstraps resampling the tree using the Neighbor-Joining algorithm with the Kimura two-parameter correction matrix. (A) *luxS* gene affiliated to *Proteobacteria*. (B) *luxS* gene affiliated to *Epsilonproteobacteria*. (C) Localizations of *R*. *exoculata* on hydrothermal vents and studied areas; red: Rainbow, green: TAG, blue: Snake Pit, orange: Logatchev (modified from [71]). Clone numbers are indicated between brackets.

**Fig 6:**
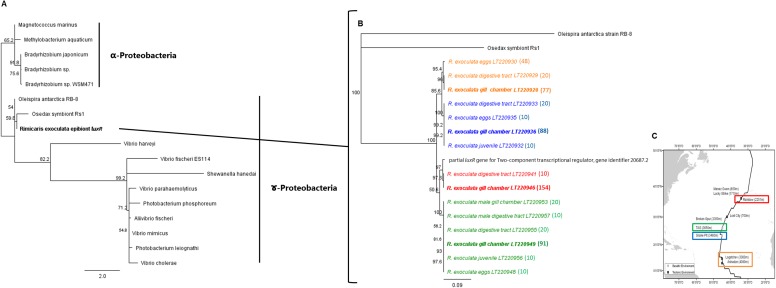
*luxR* gene phylogeny (calculated on 560 bp) of symbionts associated with the shrimp *R*. *exoculata*. The robustness was tested using 500 bootstraps resampling the tree using the Neighbor-Joining algorithm with the Kimura two-parameter correction matrix. (A) *luxR* affiliated to *Proteobacteria*. (B) *luxR* affiliated to *Gammaproteobacteria*. (C) Localizations of *R*. *exoculata* on hydrothermal vents and studied areas; red: Rainbow, green: TAG, blue: Snake Pit, orange: Logatchev (modified from [71]). Clone numbers are indicated between brackets.

The phylogenetic trees of the *luxS* and *luxR* gene sequences are presented in Figs 5 and 6. Nucleotide sequences of the *luxS* gene revealed the presence of three distinct clades according to the three vent site origins (Rainbow, TAG and Snake Pit). All the *luxS* gene sequences (378 cloned sequences, Table 4) were affiliated to the *Epsilonproteobacteria Sulfurovum* sp. NBC37-1 (86% similarity), the closest symbiont lineage relative (Fig 5). Nucleotide sequence analysis of the *luxR* gene revealed the presence of four distinct clades, each associated with a single vent site. All the *luxR* gene sequences (608 cloned sequences) were affiliated to *Gammaproteobacteria* (77% to 80% similarity) (Fig 6). According to the phylogenetic analysis, the *luxS* gene from the *Epsilonproteobacteria* epibiont is more divergent than the *luxR* gene from *Gammaproteobacteria*, and even still more divergent than the *16S* [24], [26]. *LuxS* is not amplified for Logatchev adults, which may be due to the gene divergence impairing primer hybridization. Regarding *luxR*, all samples including eggs and juveniles were successfully amplified. It should be noted that eggs and juveniles have the same *luxR* sequence as adults collected at the same site. This indicates that recently recruited juveniles had either, i) hatched at the same location where they had been sampled (no dispersal event) or ii) acquired the hydrothermal vent selected epibiontic microbial community after a molt event that followed recruitment.

Until now, based on *16S* rDNA analyses, gill chamber symbiont transmission at each molt cycle has been supposed to take a horizontal pathway [24]. According to the host genetic markers [4–5], only one host haplotype can be identified along the MAR, suggesting a single host population. The gut microbial community *16S* rDNA diversity is always restricted to four lineages: *Gammaproteobacteria* (one OTU related to *Leucothrix mucor*), *Epsilonproteobacteria* (clusters epsiA and espiB related to *Sulfovorum* sp.), Mollicutes (groups A, B, C and D) and Deferribacteres (single OTU) [14]. According to the gut symbiont *16S* rRNA sequence analyses for these four gut lineages, segregations start to appear among sites from north (Rainbow) to south (Logatchev), mostly for the *Gammaproteobacteria* and *Epsilonproteobacteria*, which are supposedly ingested by the shrimp [13–14]. Until now, however, *16S* rDNA approaches have led to partial conclusions, as these sequences are insufficiently divergent and did not show a complete segregation between sites [14], [24].

Here, using the *lux* gene analyses, we observed clear patterns of distribution consistent with geographical patterns. Our results suggest that geographical isolation must be considered as a factor acting upon *lux* genetic variations within and among *R*. *exoculata* epibiont populations. So, clades observed here for each *lux* gene could be considered as geotypes [72–73], having the best fitness with regards to the constrains of the geochemical sites. Two hypotheses can therefore be proposed to explain this microdiversity among the *lux* genes that seems to be linked to the site of shrimp origin. The first hypothesis would be that the populations of symbionts are genetically isolated because of gene flux barriers between distantly related hydrothermal sites. In this model, events of dispersion would be rare or absent and the gene fluxes within the symbiotic community on a particular site would be greater than inter-site fluxes responsible for the mixing of communities. If this is the case, then symbiont microdiversity reflects the diversity of the local microbial community [24], adapted to its local environment. It has also been shown that biogeography plays a major role in the structuring of other symbiotic bacterial populations in the northern MAR hydrothermal sites [74]. A second hypothesis postulates that the populations of symbionts are not completely spatially structured along the MAR, so all symbionts would be found everywhere [24]. For example, free-living symbiotic forms at the Rainbow site would also be present at the TAG site and *vice versa*. Under this hypothesis, the structuring of the symbiotic populations of each site would be due to the colonization of the hosts by their selected symbionts issued from a free-living pool after recruitment. This model therefore implies the existence of highly specific mechanisms of communication and recognition between the hosts and their symbionts among all those present, symbionts that would be selected for their fitness. Moreover, a recent study on the *luxS* gene among *Epsilonproteobacteria* in a deep-sea vent [63], showed that bacteria inhabiting similar ecological niches, regardless of their taxonomic distance, show closely related forms of the *luxS* gene. This would be observed because habitat and ecological niche play an important role in population selection, based on the ability to communicate. Interspecies QS within the same habitat and niche would therefore be an important driver of *luxS* evolution. To go further, more locations need to be visited and more samplings of free larvae would need to be made to draw clear conclusion about epibiont recruitment.

Our sequences were clearly clustered as a single clade per site. This could be linked to different factors: the geochemistry of the site (*i*.*e*. basaltic *vs* ultramaphic), the depth, and the geographical location (from north to south) [1], [75]. However, Petersen and colleagues showed that, despite a predominance of bacteria affiliated to a single *Epsilonproteobacteria* lineage, whatever the hydrothermal site considered, microdiversity was observed according to site of origin and, probably, geochemistry [24]. In our study, *luxS* genes showed a proximity between the two basaltic sites relative to the ultramaphic Rainbow (Fig 5), while *luxR* genes (Fig 6) clustered Logatchev with Snake Pit (*i*.*e*. geographical) rather than with Rainbow (*i*.*e*. geochemistry of ultramaphic sites). Drawing clear conclusions about the relative effects of chemistry *vs* geography would require samples from a greater number of vent locations showing contrasting geochemical conditions.

### Concluding remarks

The presence of the *lux* genes in the epibiontic community of *R*. *exoculata* at different molt and life stages was confirmed for the Rainbow, TAG, Snake Pit and Logatchev vent sites. Whatever the point in the life cycle, from eggs (only *luxR*) to the adults, or in the molt cycle, *luxS* or *luxR* gene phylogenetic analyses clustered the bacteria in a single clade per site of origin. This makes *lux* genes good candidates for biogeographical purposes, *luxR* being more accurate. These results also indicate that eggs are colonized by epibionts from the adults of the same location and confirm the epibiontic population shift toward G*ammaproteobacteria* (*luxR* gene analyses Fig 6). Juveniles studied here were already recruited among adult aggregates and had the same epibiontic population as the adults of the same site. Therefore, recruited juveniles might i) be recruited from the larval pool of the same site without a migration event or ii) would have migrated and subsequently rid themselves of their original epibiont population (during a molt event, for example) and be already newly colonized by the local epibiont population with better fitness. To decide between these two hypotheses, samples from free larvae collected from the bottom seawater will need to be amplified and compared. Finally, RT-PCR experiments revealed gene expression and thus potential QS activity of epibiont shrimps from the TAG, Snake Pit and Rainbow deep sea hydrothermal vent sites. Functions associated with biofilm formation in pathogenic *Epsilonproteobacteria* are regulated by the AI-2 signal [48], [60], [76], [77], [78], [79], [80]. We can therefore hypothesize that *R*. *exoculata* gill chamber colonization by the epibiont community at each molt could be sustained by AI-2 and several LuxR solo proteins that could intercept this QS signal as well as compounds from the host. Future efforts leading to the development of a genetic system in vent *Proteobacteria* would help us to better understand the *luxS* / AI-2 QS system in hydrothermal environments.

## Supporting information

S1 File The SI File is a more detailed description of bioinformatic studies followed by extraction and quantification of N-acylhomoserine lactones (AHLs) description. (DOC)

S1 Fig LuxS protein sequences alignment. The words red/black, orange and white, are respectively used to describe the microbial mat at the end, intermediate stage, and beginning of the molt cycle. Black boxes indicate similarity of amino acids sequences. The red square shows a region necessary for the enzyme activity that is conserved in all *luxS* gene.
(TIF)

S2 Fig LuxR protein analysis. (A) LuxR protein sequence alignment. The words red/black and white are used to describe the microbial mat at the end and beginning of the molt cycle, respectively. Black boxes indicate similarity of amino acid sequences. LuxR type receptors share a modular domain structure, with a N-terminal signal binding domain (SBD) and a C-terminal DNA binding domain (DBD) with the conserved “HTH LUXR” motif (yellow hexagon). The N-terminus is marked with an N and the C-terminus with a C. LuxR were identified using BLAST [15] software and SMART 7 software [16]. (B) Conserved amino acid motifs of LuxR-type proteins from *Rimicaris exoculata* epibionts. **Upper part**: Motif of the six conserved amino acid positions in typical AHL sensors. Protein sequences of luxR from *Vibrio fischeri*, TraR from *Agrobacterium tumefaciens*, SdiA from *Escherichia coli*, QscR and LasR from *Pseudomonas aeruginosa* were used to generate the alignment [17]. **Lower part**: Motif of the six conserved amino acids of LuxR from *Rimicaris exoculata* epibionts. All alignments were generated with Geneious software. The sequence logo was made with WebLogo3 [18].
(TIF)

S3 Fig Chromatograph of *N*-acylhomoserine lactone extraction. (A) C_4_-AHL standard (1) and 3-oxo-C_12_-HSL standard (2). (B) branchiostegite and C_4_-AHL extraction control (3). (C) and (F) scaphognathite. (D) abdomen and C_4_-AHL extraction control (4). (E) branchiostegite. (G) abdomen.
(TIF)
