# Supplementary material for: Highlighting of quorum sensing lux genes and their expression in the hydrothermal vent shrimp Rimicaris exoculata ectosymbiontic community. Possible use as biogeographic markers
Source: PLoS One. 2017 Mar 22;12(3):e0174338. doi: 10.1371/journal.pone.0174338 (PMC5362221; doi:10.1371/journal.pone.0174338)
Supplement: S1 File — (DOC) [file pone.0174338.s001.doc]

**Supporting Information**

**Bioinformatic studies**

*In silico*, the LuxS protein sequence alignment shows three groups, which differ according to site (S1 Fig.). Amino acid variations between these three groups in positions 2, 11, 13, 29, 31, 32, 128, 132, 133, 134, 135, and 144 does not seem to indicate any particular tendency. Translated sequences revealed that the *luxS* gene found here has a conserved domain common to the *luxS* genes found in other species, which is essential for LuxS enzyme activity (S1 Fig., site 41-45, HTLEH) [1]. This distribution is valid whatever the colonized location (Br or Sc) and molt stage considered. Transcripts related to *Sulfurovum* and *Sulfurimonas* spp. were detected in a low temperature biofilm of an active vent, and the expression of these genes was also correlated with AI-2 activities and with a QS response in *V. harveyi* [2]. This implies that AI-2 signals can be produced *in situ*. As epibionts of *R. exoculata* are still not cultivable, no biochemical approaches could be used in this study. However, *luxS* gene transcripts were correctly amplified (RT-PCR) and the LuxS protein sequence is conserved and seems to be able to produce AI-2. Therefore, it is possible that at least some *Epsilonproteobacteria* epibionts would have AI-2 activity during the shrimp colonization processes. Indeed, luxS/AI-2 is conserved among *Epsilonproteobacteria* [2] which is the main phylotype found in the gill chamber epibiont community here. Moreover, despite QS signaling molecules, such as acylhomoserine lactones that appear to have inherent susceptibility to hydrolytic degradation, the precursor of AI-2 (DPD) is very stable over a broad pH range [3]. In line with this hypothesis, Nichols and colleagues demonstrated AI-2 production in the vent hyperthermophiles *Thermotoga maritima* and *Pyrococcus furiosus* grown in laboratory, via a *luxS*-independent biotic / abiotic reaction pathway [4]. This study showed the stability of AI-2 at elevated temperature. So it is possible that the epibiont community of the gill chamber, living in environments characterized by harsh physicochemical conditions, communicated using AI-2, but as epibionts are not yet grown under laboratory condition, experiment to detect AI-2 could not be undertaken.

The LuxR protein alignment showed a C-terminal DNA-binding domain (DBD) with a helix- turn-helix motif, the “HTH LUXR” motif and an N-terminal signal-binding domain (SBD) connected with a short linker (S2A Fig). These two functional domains are found in LuxR-type receptors [5]. Moreover, RT PCR experiments gave expected size transcripts indicating expression of *lux*R genes. Usually, LuxR-type protein-sensing AHLs harbor six highly conserved amino acids in the N-terminus SBD that are important for signal-binding, signal molecule specificity and shaping the signal-binding pocket. These six conserved amino acids are W57, Y61, D70, P71, W85 and G113 (with respect to *Agrobacterium* sp. TraR, S2B Fig.), which are either hydrophobic or aromatic, displaying a conserved motif for AHL-sensors [6]. Bioinformatic analyses of *R. exoculata* epibiont LuxR amino acids at these positions revealed that the conserved WYDPWG-motif of the AHL-sensors was absent (S2B Fig.). However, the N-terminus SBD can harbor diverse domains that determine the signal-sensing specificity of the receptor [7]. The variability of the SBD domains enables bacteria to sense diverse signals, like exogenous AHLs, exogenous or endogenous non-AHLs, or eukaryotic signals, and can thereby influence different bacterial processes [8]. Since no *luxI* has yet been found in the epibiont metagenome [9], and no AHLs could be detected in our study (S3 Fig.), it is possible that these LuxR protein would be LuxR solos [8], [10], [11]. LuxR solos form a protein family highly similar to QS LuxRs, which does not possess an associated cognate LuxI protein. A recent study in the insect pathogen *Photorhabdus luminescens* found some LuxR solos, designated PluR, which respond to a new class of endogenously produced molecules, the α-pyrones, which are not AHLs [12]. Moreover, a LuxR solo sub-family has been discovered that is only found in plant-associated bacteria that do not bind AHLs but instead bind plant produced compounds [11], [13], [14].

**S1 Fig. LuxS protein sequences alignment.**

The words red/black, orange and white, are respectively used to describe the microbial mat at the end, intermediate stage, and beginning of the molt cycle. Black boxes indicate similarity of amino acids sequences. The red square shows a region necessary for the enzyme activity that is conserved in all *luxS* gene.

**S2 Fig. LuxR protein analysis.**

(A) LuxR protein sequence alignment. The words red/black and white are used to describe the microbial mat at the end and beginning of the molt cycle, respectively. Black boxes indicate similarity of amino acid sequences. LuxR type receptors share a modular domain structure, with a N-terminal signal binding domain (SBD) and a C-terminal DNA binding domain (DBD) with the conserved “HTH LUXR” motif (yellow hexagon). The N-terminus is marked with an N and the C-terminus with a C. LuxR were identified using BLAST [15] software and SMART 7 software [16]. (B) Conserved amino acid motifs of LuxR-type proteins from *Rimicaris exoculata* epibionts. **Upper part**: Motif of the six conserved amino acid positions in typical AHL sensors. Protein sequences of luxR from *Vibrio fischeri*, TraR from *Agrobacterium tumefaciens*, SdiA from *Escherichia coli*, QscR and LasR from *Pseudomonas aeruginosa* were used to generate the alignment [17]. **Lower part**: Motif of the six conserved amino acids of LuxR from *Rimicaris exoculata* epibionts. All alignments were generated with Geneious software. The sequence logo was made with WebLogo3 [18].

**S3 Fig. Chromatograph of *N*-acylhomoserine lactone extraction.**

1. C_4_-AHL standard (1) and 3-oxo-C_12_-HSL standard (2). (B) branchiostegite and C_4_-AHL extraction control (3). (C) and (F) scaphognathite. (D) abdomen and C_4_-AHL extraction control (4). (E) branchiostegite. (G) abdomen.

Several results demonstrate that bacterial QS signals can also be sensed by eukaryotic organisms. For instance, using AHL-producing and AHL-nonproducing *V. anguillarum* strains, the zoospores of the green alga of the genus *Enteromorpha* attach themselves to AHL producing bacteria biofilms [19]. A similar result has been reported for the zoospores of macroalgae of the genus *Ulva* [20] and cypris larvae of the barnacle *Balanus improvisus* [21], which are attracted to bacterial biofilms and preferentially settle on those of AHL-producing bacteria. Interkingdom communication via the QS can also be bidirectional. Indeed, Wu and colleagues showed that human stress molecules can be recognized by the QS system of *Pseudomanas aeruginosa* strains and can activate QS-regulated virulence genes [22]. Studies involving animals have only described responses to OC_12_-HSL, which induces an increased synthesis of IL-8 in human lung fibroblasts [23] and the acceleration of apoptosis in macrophages and neutrophils [24]. Data is still lacking on the perception of other AHL by animals [25]. This contrasts with the situation described for plants, which respond differentially to various AHLs and which, unlike animals, are not dependent upon the integrity of the AHL molecules. Finally, eukaryotes can produce compounds referred to as Quorum Quenching (QQ) actors, which interact directly with the compounds of bacterial QS to induce signal disturbance [26], [27], [28]. This indicates that a communication system between microorganisms and eukaryotes may exist at the cellular level, therefore, *R. exoculata* and its symbionts could communicate via the QS.

**Extraction and quantification of *N-*acylhomoserine lactones (AHLs)**

**Extraction of AHLs**

The AHL extraction protocol was adapted from Morin [29]. Internal membranes of branchiostegites, scaphognathites, exopodites, abdomens and guts of frozen and immersed *R. exoculata* (adult specimens of 3 to 3.5 cm long) were dissected and each part was weighed. The samples were ground in a FastPrep bead beater (10 sec, power 4, 4°C) in 700 µL of phosphate buffered saline-sea water using a microbead matrix and centrifuged (2 min, 8000 g, 4°C). For each sample, the supernatants were recovered and then combined and centrifuged (10 min, 10,000 rpm, 4°C) to remove cell debris. The clear supernatant was recovered and taken up in 20 ml of dichloromethane. The mixture was shaken by inversion for 3 minutes and the phases then separated by centrifugation (10 minutes, 4000 g, 4°C). The lower organic phase was recovered and a second extraction of the remaining aqueous phase was then carried out. The collected dichloromethane extracts were then combined, dried over anhydrous magnesium sulfate (MgSO_4_), filtered and evaporated to dryness at 30°C (Rotavapor, Buchi, Switzerland). Residues were dissolved in 1 mL of HPLC-grade acetonitrile (Carlo Erba), and filtered through a 0.45 µm PTFE syringe filter (Alltech, France). The sample was then stored for 24 to 48 hours, protected from light at -20°C, until analysis. All samples were stored within 48 hours of extraction.

**Mass spectrometry analysis**

Mass spectrometry analysis was performed by on-line LC-MS-MS [94]. Solutions of *N*-acyl or oxo-acylhomoserine lactones of 1, 2 and 5 mg / ml were used as internal standards only. The LC-separated compounds were detected by electrospray ionization ion trap mass spectrometry (ESI-MS) using a Bruker Esquire-LC spectrometer (Bruker Daltonic, Germany) under positive-ion conditions. The software used was Bruker Esquire-LC NT version 6.08 and Agilent Technologies ChemStation.

**AHL study**

The *luxI* gene was not recovered in previous attempts using metagenomic approaches [9]. However, as the metagenome is still incomplete, *luxI* might be present but not yet sequenced. To test this hypothesis, several AHL extractions were done in this present study. To test the effectiveness of extraction, a standard control (C_4_-AHL) (2 µg / mL) was added to the sample. To test the effect of the waiting time on ice and light exposure, an inherent part of dissection of animals on board the vessel, a standard control was added at the time of dissection of Br and at the time of grinding muscles. Extractions were done in order to optimize the yield of AHLs at several key stages of the procedure. (i) Extractions were performed on several types of sample. An extraction was done on 15 shrimps per site (Rainbow, TAG and Snake Pit) at the beginning of a molt cycle and stored in dichloromethane at -20°C. At the beginning of the cycle, the epibiont community was little developed. Extractions were then performed again on Rainbow shrimps using 30 shrimps to provide a sufficient quantity of symbionts for the AHL extraction. The same extraction was performed on shrimp from the Rainbow, TAG and Snake Pit sites at the end of the molt cycle, these specimens having been stored alive in dichloromethane at -20°C once aboard. Specimens at the end of the molt cycle provide a larger quantity of symbionts for AHL extraction. Another extraction was done on the whole gill chamber of 45 entire shrimps from the Rainbow site at the end of their molt cycles and stored at -20°C. As the gill chamber is almost closed, the liquid contained in the head, in which epibionts bathe, is mostly conserved during the freezing of the shrimp. Therefore, an extraction on the whole head should have allowed possible AHLs present in “the supernatant of the gill chamber” to be recovered. (ii) The recovery of AHLs in the solvent was done in two stages to optimize the recovery of all forms of AHLs: a first time with neutral pH, then a second time after aqueous stage acidification by the addition of chloric acid to favor the recovery of acid AHLs. (iii) The filtering of MgSO_4_ was done with a glass fiber filter instead of a paper filter, as used previously, to avoid AHLs sticking to the filter. During the LC-MS-MS analysis, standard solutions of AHLs of known concentration were first injected, eluted in liquid chromatography and then passed through the mass spectrometer for calibration. Then solutions of extracted AHLs were analyzed in LC-MS-MS. The retention time and MS-MS spectra were compared with standard ones. Chromatographs were similar for all extractions (S3 Fig.). Spectrum B and D samples containing the control of extraction showed the presence of a peak in a retention time of 10 minutes corresponding to that of C_4_-HSL (spectrum A). The areas under peak of the B and D spectra reveal a loss of one sixth of C_4_-HSL during extraction if compared with area under peak of the standard passed alone (spectrum A). Sample spectra showed no specific retention time corresponding to the AHLs targeted (S3 Fig.). M/z reports recorded after division gave readings of 102, but no m/z reports corresponded to acyl chains of AHLs. A search was performed on all the m/z of the AHL family in MS-MS. M/z report peaks recorded at 102 did not correspond to a known retention time. Despite all our attempts, no AHLs could were ever revealed during this study. Several hypotheses can be suggested. Firstly, this absence could be explained by quantities of AHLs in the sample being under the limit of the apparatus detection level (2 ng / µL). Secondly, AHLs can undergo chemical degradation opening the lactone ring by addition of a water molecule. This phenomenon, called lactonolysis, occurs spontaneously in aqueous solutions [30], [31]. It is strongly favored at high temperature and under alkaline pH, and can be reversed in acidic pH solutions. Thirdly, as symbionts still cannot be cultured, the trials for extractions were done on intracellular AHLs, which are rapidly excreted into the surrounding seawater. So the concentration of intracellular AHLs is weaker than the extracellular concentration, although the latter would be difficult to collect using our approach, even with whole shrimp heads. Fourthly, sampling and shrimp recovery on board is a possible cause of stress (variation in pressure and temperature) for the epibionts, which could produce less AHLs as a result. Moreover, the conservation of shrimps in dichloromethane at -80°C over long periods may not be optimal for AHL preservation. The freezing / defrosting could also cause deterioration of AHLs. Long chain AHLs are less prone to degradation than short-chain AHLs [31]. The half-life of N-hexanoyl-homoserine lactone (C_6_-HSL) varies from over 21 days (pH 5.5, 4◦C) to less than 30 min (pH 8.5, 37°C) [30], [32]. Fifthly, the gill chamber could contain compounds that might interact negatively with AHLs, such as inhibitors [26], [27], [28]. Finally, *luxI* may not be present in the symbiotic population at all. All these reasons could contribute to explaining why AHLs were not detected in the epibiont community of the gill chamber.

## References

1. De Keersmaecker SCJ, Sonck K, Vanderleyden J. Let LuxS speak up in AI-2 signaling. TRENDS in Microbiology. 2006;14: 114-119.
2. Perez-Rodríguez I, Bolognini M, Ricci J, Bini E, Vetriani C. From deep-sea volcanoes to human pathogens: a conserved quorum-sensing signal in *Epsilonproteobacteria*. ISME J. 2015;9(5): 1222-34.
3. Globisch D, Lowery CA, McCague KC, Janda K. Uncharacterized 4,5-dihydroxy-2,3-pentanedione (DPD) molecules revealed through NMR spectroscopy: implications for a greater signaling diversity in bacterial species. Angew Chem Int Ed. 2012;51: 4204–4208.
4. Nichols JD, Johnson MR, Chou CJ, Kelly RM. Temperature, not LuxS, mediates AI-2 formation in hydrothermal habitats. FEMS Microbiol Ecol. 2009;68: 173–181.
5. Nasser W, Reverchon S. New insights into the regulatory mechanisms of the LuxR family of quorum sensing regulators. Anal. Bioanal. Chem. 2006;387: 381–390.
6. Patankar AV, González JE. Orphan LuxR regulators of quorum sensing. FEMS Microbiol. Rev. 2009;33: 739–756.
7. Skerker JM, Prasol MS, Perchuk BS, Biondi EG, Laub MT. Two-component signal transduction pathways regulating growth and cell cycle progression in a bacterium: a system-level analysis. PLoS Biol. 2005;3(10):e334.
8. Subramoni S, Venturi V. LuxR-family “solos”: bachelor sensors/regulators of signalling molecules. Microbiology. 2009;155: 1377–1385.
9. Schmidt C, Vuillemin R, Le Gall C, Gaill F, Le Bris N. Geochemical energy sources for microbial primary production in the environment of hydrothermal vent shrimps. Mar. Chem. 2008;108: 18-31.
10. Fuqua C. The QscR Quorum-Sensing Regulon of *Pseudomonas aeruginosa*: an Orphan Claims Its Identity. J Bacteriol. 2006; 188(9): 3169–3171.
11. González JF, Venturi V. A novel wide spread interkingdom signaling circuit. Trends Plant Sci. 2013; 18: 167–174.
12. Brachmann AO, Brameyer S, Kresovic D, Hitkova I, Kopp Y, Manske C, et al. Pyrones as bacterial signaling molecules. Nat.Chem.Biol. 2013;9: 573–578.
13. Patel HK, Suárez-Moreno ZR, Degrassi G, Subramoni S, González JF, Venturi V. Bacterial LuxR solos have evolved to respond to different molecules including signals from plants. Front Plant Sci. 2013;4: 447.
14. Venturi V, Fuqua C. Chemical signaling between plants and plant-pathogenic bacteria. Annu Rev Phytopathol. 2013;51: 17-37
15. Altschul SF, Gish W, Miller W, et al. Basic local alignment search tool. J. Mol. Biol. 1990;215: 403-410.
16. Letunic L, Doerks T, Bork P. SMART 7: recent updates to the protein domain annotation resource. Nucleic Acids Res. 2012 doi: 10.1093/nar/gkr931.
17. Brameyer S, Kresovic D, Bode HB, Heermann R. LuxR solos in *Photorhabdus* species. Front Cell Infect Microbiol. 2014;4: 166.
18. Crooks GE, Hon G, Chandonia JM, Brenner SE. WebLogo: a sequence logo generator. Genome Res. 2004;14(6):1188-90.
19. Joint I, Tait K, CallowME, et al*.* Cell-to-cell communication across the prokaryote-eukaryote boundary. Science. 2002;298: 1207.
20. Tait K, Joint I, Daykin M, et al*.* Disruption of quorum sensing in seawater abolishes attraction of zoospores of the green alga *Ulva* to bacterial biofilms. Environ Microbiol. 2005;7: 229–40.
21. Tait K, Havenhand J. Investigating a possible role for the bacterial signal molecules N-acylhomoserine lactones in *Balanus improvisus* cyprid settlement. Mol Ecol. 2013;22: 2588–602.
22. Wu L, Estrada O, Zaborina O, Bains M, Shen L, Kohler JE, et al. Recognition of host immune activation by *Pseudomonas aeruginosa*. Science. 2005;309(5735): 774-7.
23. Smith RS, Fedyk ER, Springer TA, Mukaida N, Iglewski BH, Phipps RP. IL-8 production in human lung fibroblasts and epithelial cells activated by the *Pseudomonas* autoinducer N-3-oxododecanoyl homoserine lactone is transcriptionally regulated by NF-kappa B and activator protein-2. J Immunol. 2001;167: 366–74.
24. Tateda K, Ishii Y, Horikawa M, Matsumoto T, Miyairi S, Pechere JC, Standiford TJ, Ishiguro M, Yamaguchi K. The *Pseudomonas aeruginosa* autoinducer N-3-oxododecanoyl homoserine lactone accelerates apoptosis in macrophages and neutrophils. Infect Immun. 2003;71: 5785–93.
25. Hughes DT and Sperandio V. Inter-kingdom signalling: communication between bacteria and their hosts. Nat Rev Microbiol**.** 2008; 6: 111–20.
26. Shiner E, Rumbaugh K, Williams S. Interkingdom signaling: Deciphering the language of acyl homoserine lactones. FEMS Microbiol. 2005;29: 935-947.
27. Grandclément C, Tannières M, Moréra S, Dessaux Y, Faure DD. Quorum quenching: role in nature and applied developments. FEMS Microbiol Rev. 2015;40(1): 86-116.
28. Dong YH, Wang LH, Xu JL, Zhang HB, Zhang XF, Zhang LH. Quenching quorum-sensing dependent bacterial infection by an N-acyl homoserine lactonase. Nature. 2001;411: 813-7.
29. Morin D, Grasland B, Vallée-Réhel K, Dufau C, Haras D. On-line high-performance liquid chromatography–mass spectrometric detection and quantification of N-acylhomoserine lactones, quorum sensing signal molecules, in the presence of biological matrices. Journal of Chromatography A. 2003;1002: 79–92.
30. Byers JT, Lucas C, Salmond GPC, Welch M. Nonenzymatic turnover of an *Erwinia carotovora* quorum-sensing signaling molecule. J Bacteriol. 2002;184: 1163–1171.
31. Yates EA, Philipp B, Buckley C, Atkinson S, Chhabra SR, Sockett RE, et al. N-acylhomoserine lactones undergo lactonolysis in a pH-, temperature-, and acyl chain length-dependent manner during growth of *Yersinia pseudotuberculosis* and *Pseudomonas aeruginosa*. Infect Immun. 2002;70(10): 5635-46.
32. Delalande L, Faure D, Raffoux A, Uroz S, D'Angelo-Picard C, Elasri M, et al. N-hexanoyl-L-homoserine lactone, a mediator of bacterial quorum-sensing regulation, exhibits plant-dependent stability and may be inactivated by germinating *Lotus corniculatus* seedlings. FEMS Microbiol Ecol. 2005;52(1):13-20.
