# Supplementary figures and images for: Highlighting of quorum sensing lux genes and their expression in the hydrothermal vent shrimp Rimicaris exoculata ectosymbiontic community. Possible use as biogeographic markers
Source: PLoS One. 2017 Mar 22;12(3):e0174338. doi: 10.1371/journal.pone.0174338 (PMC5362221; doi:10.1371/journal.pone.0174338)

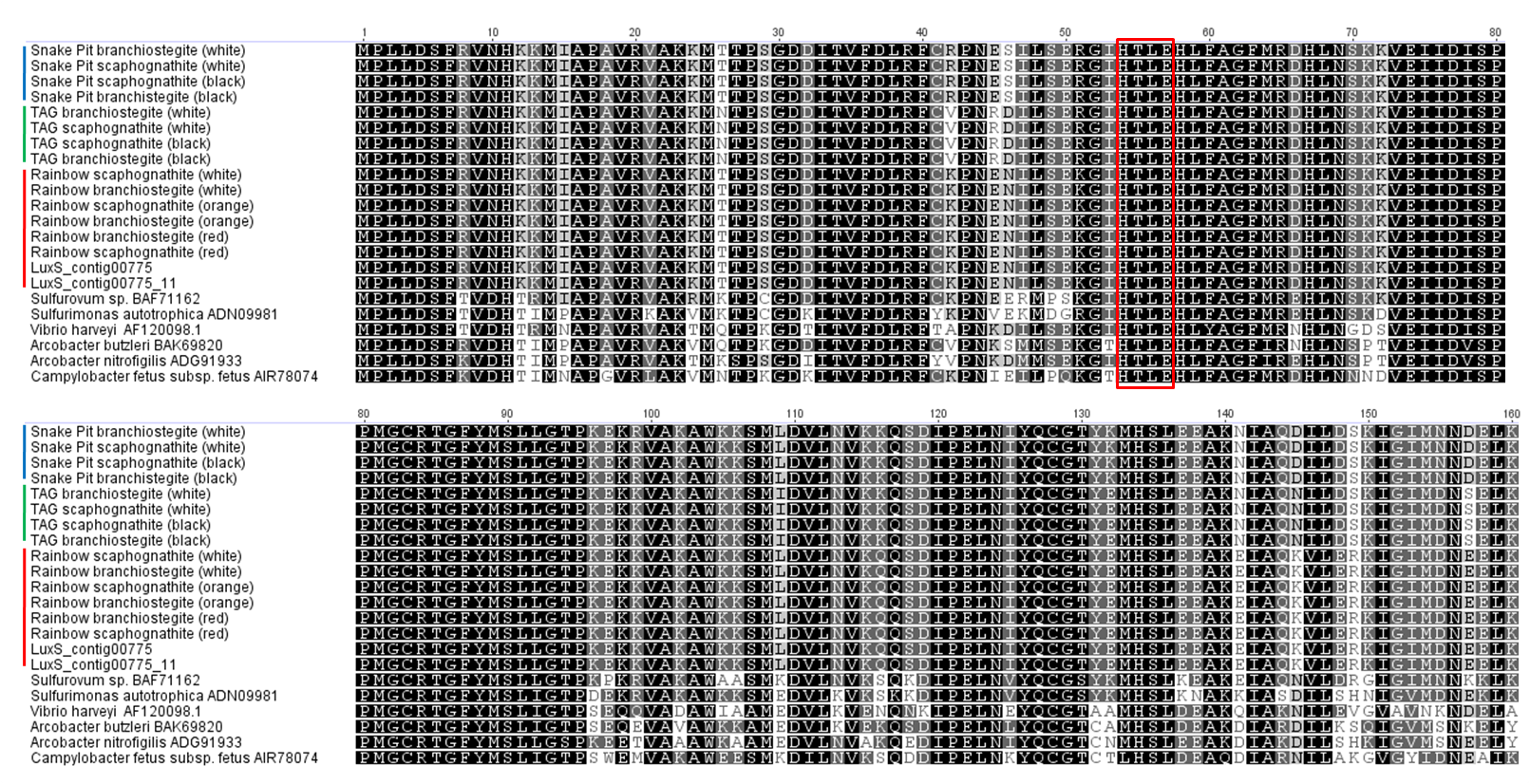

Supplement: S1 Fig — The words red/black, orange and white, are respectively used to describe the microbial mat at the end, intermediate stage, and beginning of the molt cycle. Black boxes indicate similarity of amino acids sequences. The red square shows a region necessary for the enzyme activity that is conserved in all luxS gene. (TIF) [file pone.0174338.s002.tif]

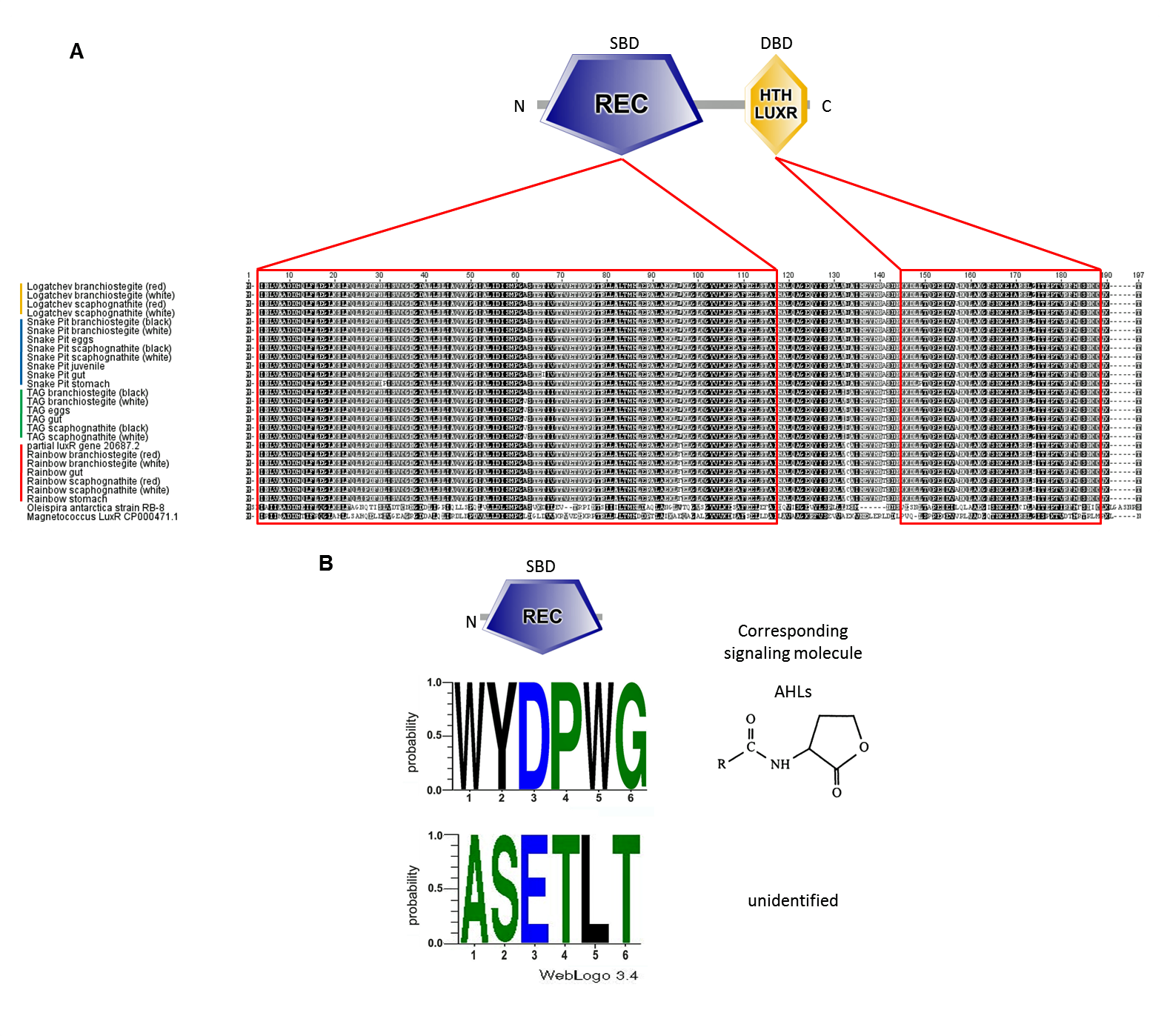

Supplement: S2 Fig — (A) LuxR protein sequence alignment. The words red/black and white are used to describe the microbial mat at the end and beginning of the molt cycle, respectively. Black boxes indicate similarity of amino acid sequences. LuxR type receptors share a modular domain structure, with a N-terminal signal binding domain (SBD) and a C-terminal DNA binding domain (DBD) with the conserved “HTH LUXR” motif (yellow hexagon). The N-terminus is marked with an N and the C-terminus with a C. LuxR were identified using BLAST [15] software and SMART 7 software [16]. (B) Conserved amino acid motifs of LuxR-type proteins from Rimicaris exoculata epibionts. Upper part: Motif of the six conserved amino acid positions in typical AHL sensors. Protein sequences of luxR from Vibrio fischeri, TraR from Agrobacterium tumefaciens, SdiA from Escherichia coli, QscR and LasR from Pseudomonas aeruginosa were used to generate the alignment [17]. Lower part: Motif of the six conserved amino acids of LuxR from Rimicaris exoculata epibionts. All alignments were generated with Geneious software. The sequence logo was made with WebLogo3 [18]. (TIF) [file pone.0174338.s003.tif]

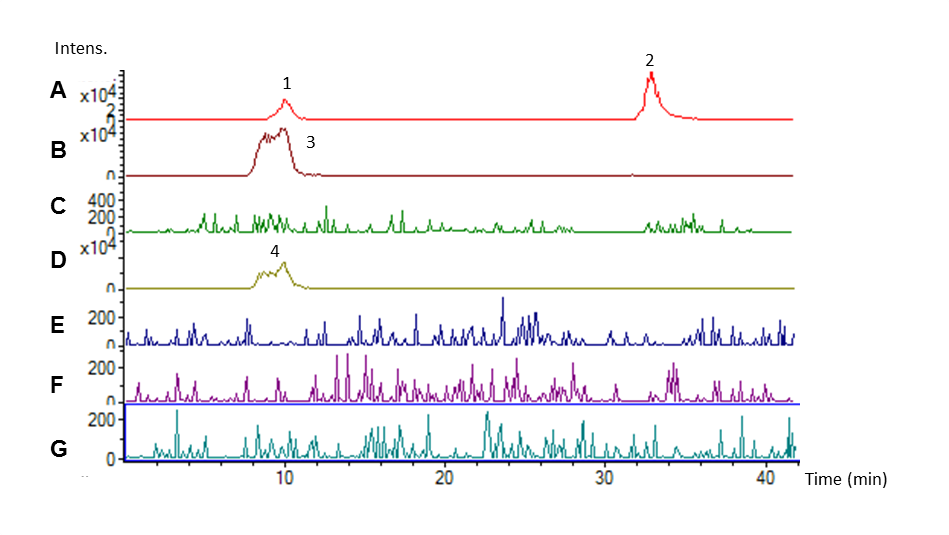

Supplement: S3 Fig — (A) C4-AHL standard (1) and 3-oxo-C12-HSL standard (2). (B) branchiostegite and C4-AHL extraction control (3). (C) and (F) scaphognathite. (D) abdomen and C4-AHL extraction control (4). (E) branchiostegite. (G) abdomen. (TIF) [file pone.0174338.s004.tif]
